# Long‐term survivor of pulmonary combined large cell neuroendocrine carcinoma treated with nivolumab

**DOI:** 10.1111/1759-7714.13471

**Published:** 2020-05-07

**Authors:** Risa Oda, Katsuhiro Okuda, Yoriko Yamashita, Tadashi Sakane, Tsutomu Tatematsu, Keisuke Yokota, Katsuhiko Endo, Ryoichi Nakanishi

**Affiliations:** ^1^ Department of Oncology, Immunology and Surgery Nagoya City University Graduate School of Medical Sciences Nagoya Japan; ^2^ Department of Pathology and Molecular Diagnostics Nagoya City University Graduate School of Medical Sciences Nagoya Japan

**Keywords:** Large‐cell neuroendocrine carcinoma (LCNEC), nivolumab, rectal metastasis

## Abstract

Several authors have previously reported that patients with pulmonary combined large cell neuroendocrine cancer ( LCNEC) have a poor prognosis and there is no consensus on the treatment strategy for combined LCNEC as well as LCNEC. Here, we report the case of a long‐term survivor with pulmonary combined LCNEC. The patient was a 60‐year‐old man who underwent thoracoscopic right lower lobectomy. The final histopathology and staging of the tumor showed LCNEC combined with squamous cell carcinoma and T2aN0M0 stage IB. Multimodality treatments including chemotherapy, radiotherapy and surgery for several recurrences were performed after the pulmonary surgery. After immune checkpoint inhibitor (ICI) therapy with nivolumab, all the metastatic lesions shrunk and a partial response was maintained at five years after the first surgery. In our case, ICI after multimodality therapy combining cytotoxic anticancer drugs and radiotherapy was effective in LCNEC with metachronous multiple metastases.

**Key points:**

**Significant findings of the study:**

Immune checkpoint inhibitor after multimodality therapy combining cytotoxic anticancer drugs and radiotherapy was effective in LCNEC with metachronous multiple metastases. The patient survived over five‐years after the first surgery.

**What this study adds:**

Immune checkpoint inhibitor may be effective in some LCNEC patients.

## Introduction

The 2015 World Health Organization classification defines pulmonary large cell neuroendocrine carcinoma (LCNEC) as a high‐grade neuroendocrine carcinoma.^1^ LCNEC is an aggressive and a rare type of lung cancer that accounts for 3% of all primary lung malignancies.^2^ LCNEC sometimes contain other components, such as small cell lung cancer (SCLC), adenocarcinoma and squamous cell carcinoma. Several authors have previously reported that patients with combined LCNEC have a poor prognosis and there is no consensus on the treatment strategy for combined LCNEC as well as LCNEC.^3^, ^4^ We herein report a recurrent case of pulmonary combined LCNEC that has achieved long‐term survival with immune checkpoint inhibitor (ICI) after continuous multimodality therapy.

## Case report

**Figure 1:**
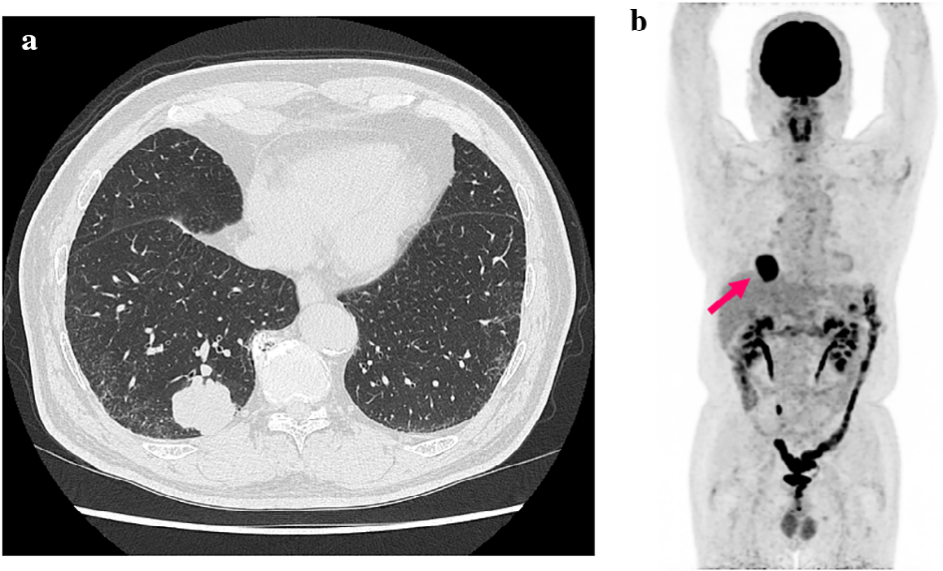
(**a**) Chest computed tomography (CT) scan revealed an abnormal mass in the right lower lobe. (**b**) ^18^F‐fluorodeoxyglucose positron emission tomography (FDG‐PET) shows a high uptake in this lesion.

A 60‐year‐old man was referred to our hospital because an abnormal shadow had been detected on his annual chest X‐ray. Chest computed tomography (CT) scan revealed a 45 mm tumor in the right lower lobe (Fig 1a). ^18^F‐fluorodeoxyglucose positron emission tomography (FDG‐PET) revealed a high uptake in this lesion (Fig 1b). The patient had a medical history of hypertension and was a current smoker (100 pack‐year). He underwent thoracoscopic right lower lobectomy with mediastinal lymph node dissection. The final histopathology and staging of the tumor showed LCNEC combined with squamous cell carcinoma and T2aN0M0 stage IB, respectively. The staining of the tumor was positive for synaptophysin (50%) and p63 (50%). Chromogranin‐A was negative. MIB‐1 index was almost 50%. Programmed death‐ligand 1 (PD‐L1) immunohistochemical (IHC) staining was not performed.

**Figure 2:**
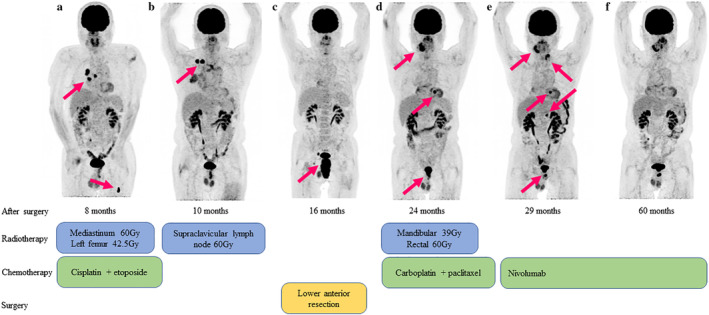
Clinical course of our case. FDG‐PET shows a high uptake in metastatic tumors (arrowhead). (**a**) Eight months after surgery; (**b**) 10 months after surgery; (**c**) 16 months after surgery; (**d**) 24 months after surgery; (**e**) 29 months after surgery; (**f**) 60 months after surgery.

Eight months after surgery, mediastinal lymph nodes and left femur metastases were found. FDG‐PET revealed a high uptake in those lesions (Fig 2a), and he subsequently received four courses of chemotherapy with cisplatin plus etoposide, 60 Gy irradiation to mediastinal lymph nodes and 42.5 Gy irradiation to his left femur. Right supraclavicular lymph node metastasis was detected (Fig 2b) 10 months after surgery, and he received 50 Gy irradiation to this lesion. A rectal tumor was detected 16 months after surgery, and was diagnosed as malignant by rectal biopsy. FDG‐PET also revealed a high uptake in the rectal tumor (Fig 2c). He underwent a lower anterior resection and it was diagnosed as metastasis from pulmonary combined LCNEC. The tumor had also spread to the right internal iliac and inguinal lymph nodes. During IHC staining of the rectal tumor, the MIB‐1 index was almost 50% the same as primary pulmonary tumor. Twenty‐four months after the first surgery, myocardial and right mandibular metastases were found. FDG‐PET revealed a high uptake in the myocardium, right mandible and rectal tumor (Fig 2d), and it was suspected that tumor had remained in the rectum. He received four courses of chemotherapy with carboplatin plus paclitaxel, 39 Gy irradiation to the right mandible and 60 Gy irradiation to the rectal tumor. Twenty‐nine months after the first surgery, a left submandibular lymph node and left adrenal metastases were newly detected (Fig 2e). He subsequently received immunotherapy with nivolumab. All the metastatic lesions shrunk and a partial response was maintained at five years after the first surgery (Fig 2f).

## Discussion

Pulmonary LCNEC is an aggressive and rare type of lung cancer.^2^ It is well known that being male, elderly and a heavy smoker are important risk factors in the etiology of LCNEC.^4^ Combined LCNEC accounts for approximately 11% of LCNEC.^3^, ^5^ Adenocarcinoma is the most common histologic component of combined LCNEC.^1^ The difference in the clinical characteristics and prognostic factors between pure LCNEC and combined LCNEC remain unclear.

No valid therapy has been proposed for the treatment of LCNEC including combined LCNEC, except for complete resection. The five‐year survival rates and distant metastasis rates of patients who received complete resection have been reported to be 15%–57% and 65%, respectively.^6^ It has been previously reported that the median overall survival of all LCNEC patients was 10.2 months.^7^ Therefore, surgery alone is insufficient to treat patients with LCNEC, and postoperative adjuvant therapy is likely to be required.^4^ There have been many reports that platinum‐ and SCLC‐based chemotherapies are effective in patients with LCNEC.^4^, ^8^ Several authors have reported that patients with LCNEC responded to nivolumab and demonstrated that immunotherapy might be effective in pulmonary LCNEC, even if PD‐L1 expression of the tumor is low.^9^, ^10^ Unfortunately, in our case, PD‐L1 expression of the tumor is unknown. The role of radiotherapy in the treatment of pulmonary LCNEC is still unclear, but some authors suggest its use in the locally advanced disease setting.^2^ Preclinical studies showed synergistic efficiency of radiotherapy combined with ICI, irrespective of tumor type.^9^ There are few previous reports of resection for metastatic tumor of LCNEC. In a case report, hepatectomy was performed for liver metastasis from pulmonary LCNEC, and there was no sign of recurrence six months after hepatectomy.^11^ Our case survived for a long‐term period because of immunotherapy with nivolumab after continuous multimodality therapy including radiotherapy and there was no regrowth of recurrent lesions for two years after the immunotherapy. This is an extremely rare case in which the patient has survived five‐years, despite having developed distant metastases eight months after the first surgery.

There is no consensus on treatment for patients with lung LCNEC. There are only few reports on the ICI outcomes in lung LCNEC and no valid data proved regarding the correlation between the molecular tumor subtype including PD‐L1 expression and response to ICIs.^9^, ^10^, ^12^, ^13^, ^14^ It is difficult to conduct prospective clinical trials because of the rarity of LCNEC. Hence, we need to continue to obtain useful information from several small‐scale studies, including case reports.

In conclusion, we report a case of LCNEC with metachronous multiple metastases in which ICI after multimodality therapy combining cytotoxic anticancer drugs and radiotherapy was effective. Although the order of each drug regimen needs further study, we believe that this report will bring hope to patients with recurrent or advanced LCNEC.

## Disclosure

The authors have no conflicts of interest to declare.
